# Supplementary material for: Diagnostic performance of the combined nasal and throat swab in patients admitted to hospital with suspected COVID-19
Source: BMC Infect Dis. 2021 Apr 6;21:318. doi: 10.1186/s12879-021-05976-1 (PMC8022129; doi:10.1186/s12879-021-05976-1)
Supplement: Supplementary file 1 — Additional file 1: eTable 1. Virology, laboratory tests at presentation with suspected COVID-19. eTable 2. Baseline characteristics of patients stratified according to whether the diagnosis of COVID-19 was confirmed or probable. eTable 3. Virology, laboratory tests at presentation stratified according to whether the diagnosis of COVID-19 was confirmed or probable. eTable 4. Use of serial testing in patients with suspected COVID-19. eTable 5. Diagnostic performance of the index and serial combined nasal and throat swab for the secondary outcome of a diagnosis of confirmed COVID-19 on serial testing. eFigure 1. Stack plot of the number of RT-PCR tests performed stratified according to whether the test was positive (red) or negative (blue). eFigure 2. Heat map of RT-PCR testing in patients with confirmed COVID-19 stratified according to whether the index test was negative (a) or positive (b). eFigure 3. Sensitivity of serial testing using the combined nasal and throat swab for the primary (confirmed and probable COVID-19) and secondary (confirmed COVID-19) outcome in patients who were tested at least four times. eFigure 4. Negative predictive value of serial testing using the combined nasal and throat swab for the primary (confirmed and probable COVID-19) and secondary (confirmed COVID-19) outcome in patients who were tested at least four times. eFigure 5. Forest plot of the (a) sensitivity and (b) negative predictive value of the index combined nasal and throat swab for a diagnosis of confirmed COVID-19 stratified by subgroups. [file 12879_2021_5976_MOESM1_ESM.docx]

DATA SUPPLEMENT

**Diagnostic performance of the combined**

**nasal and throat swab in patients**

**admitted to hospital with suspected COVID-19**

Kuan Ken Lee, M.D.,^1^ Dimitrios Doudesis, M.Sc.,^1, 2^ Daniella A. Ross, M.D.,^3^

Anda Bularga, M.D.,^1^ Claire L. MacKintosh, M.D. , Ph.D.,^3^ Oliver Koch, M.D., Ph.D.,^3^

Ingolfur Johannessen, M.D., Ph.D.,^4^ Kate Templeton, Ph.D., FRCPath.,^4^

Sara Jenks, M.D., FRCPath.,^5^ Andrew R. Chapman, M.D., Ph.D.,^1^

Anoop S.V. Shah, M.D., Ph.D.,^1,6,7^ Atul Anand, M.D., Ph.D.,^1^

Meghan R. Perry, M.D., Ph.D.,^3^ Nicholas L. Mills, M.D., Ph.D.^1, 2^

*on behalf of the DataLoch COVID-19 Collaboration**

^1^ BHF Centre for Cardiovascular Science, University of Edinburgh, Edinburgh, UK.

^2^ Usher Institute, University of Edinburgh, Edinburgh, UK.

^3^ Regional Infectious Disease Unit, Western General Hospital, Edinburgh, UK.

^4^ Department of Clinical Virology, Royal Infirmary of Edinburgh, Edinburgh, UK

^5^ Department of Clinical Biochemistry, Royal Infirmary of Edinburgh, Edinburgh, UK.

^6^ Department of Non-communicable Disease Epidemiology, London School of Hygiene and Tropical Medicine, London, UK

^7^ Department of Cardiology, Imperial College Healthcare NHS Trust, London, UK

**Corresponding Author:**

Dr Kuan Ken Lee

BHF/University Centre for Cardiovascular Science

The University of Edinburgh

Edinburgh EH16 4SA

United Kingdom

Telephone: 0044 131 242 6515

E-mail: [ken.lee@ed.ac.uk](mailto:ken.lee@ed.ac.uk)

**eTables:** 5

**eFigures:** 5

**eTable 1.** Virology, laboratory tests at presentation with suspected COVID-19

|  | **All**  (n = 1,368) | **COVID-19***  (n = 496) | **Alternative diagnosis**  (n = 872) | **P-value‡** |
| --- | --- | --- | --- | --- |
| Positive index PCR test | 259 (19%) | 255 (51%) | 4 (0.5%)^†^ | <0.001 |
| White cell count, x10^9^/L | 8.9 [6.5-12.5] | 8.1 [6.0-11.8] | 9.4 [6.9-13.0] | <0.001 |
| Lymphocyte count, x10^9^/L | 1.32 [0.87-1.89] | 1.14 [0.78-1.61] | 1.44 [0.94-2.03] | <0.001 |
| Neutrophil count, x10^9^/L | 6.2 [4.2-9.4] | 5.8 [4.0-8.9] | 6.4 [4.4-9.9] | <0.001 |
| C-reactive protein, mg/dL | 32 [7-105] | 52 [13-123] | 22 [6-94] | <0.001 |
| ALT, IU/L | 20 [13-33] | 22 [14-36] | 18 [13-32] | <0.001 |
| Impaired renal function | 398 (30%) | 158 (32%) | 240 (28%) | 0.11 |

Values are No. (%) or median [inter-quartile range].

Abbreviations: ALT = alanine transaminase; PCR = polymerase chain reaction

Impaired renal function = estimated glomerular filtration rate of <60 mL/min

* Confirmed or probable COVID-19 with no alternative diagnosis

† Four patients were adjudicated to be asymptomatic carriers of SARS-CoV-2

‡ Comparison between patients with COVID-19 versus those with alternative diagnosis

**eTable 2.** Baseline characteristics of patients stratified according to whether the diagnosis of COVID-19 was confirmed or probable

|  | **All**  **COVID-19**  (n = 496) | **Confirmed COVID-19**  (n = 323) | **Probable**  **COVID-19**  (n = 173) | P-value**‡** |
| --- | --- | --- | --- | --- |
| **Age,** years | 71 (57, 82) | 73 (59, 83) | 66 (52, 78) | <0.001 |
| **Sex** |  |  |  | 0.071 |
| Men | 281 (57%) | 193 (60%) | 88 (51%) |  |
| Women | 215 (43%) | 130 (40%) | 85 (49%) |  |
| **Ethnicity** |  |  |  | 0.11 |
| White | 372 (97%) | 238 (96%) | 134 (99%) |  |
| Other | 11 (2.9%) | 10 (4.0%) | 1 (0.7%) |  |
| **Deprivation**† |  |  |  | 0.044 |
| 1 (most deprived) | 65 (13%) | 32 (10%) | 33 (19%) |  |
| 2 | 116 (24%) | 81 (25%) | 35 (20%) |  |
| 3 | 79 (16%) | 51 (16%) | 28 (16%) |  |
| 4 | 93 (19%) | 66 (21%) | 27 (16%) |  |
| 5 (least deprived) | 139 (28%) | 89 (28%) | 50 (29%) |  |
| **Duration of symptoms,** days | 3.0 [2.0-7.0] | 3.0 [2.0-7.0] | 4.0 [2.0-10.0] | 0.050 |
| **Clinical features** |  |  |  |  |
| Fever | 322 (65%) | 221 (68%) | 101 (59%) | 0.040 |
| Upper respiratory tract symptoms | 63 (13%) | 36 (11%) | 27 (16%) | 0.20 |
| Lower respiratory tract symptoms | 419 (85%) | 270 (84%) | 149 (86%) | 0.59 |
| Systemic symptoms | 257 (52%) | 166 (51%) | 91 (53%) | 0.87 |
| Neurological symptoms | 118 (24%) | 78 (24%) | 40 (23%) | 0.87 |
| Gastrointestinal symptoms | 107 (22%) | 73 (23%) | 34 (20%) | 0.51 |
| Lymphopenia | 406 (82%) | 277 (87%) | 129 (75%) | 0.001 |
| Inflammation | 421 (86%) | 287 (90%) | 134 (77%) | <0.001 |
| Radiological signs | 316 (64%) | 216 (68%) | 100 (58%) | 0.037 |
| **Comorbidities** |  |  |  |  |
| Diabetes mellitus | 96 (19%) | 65 (20%) | 31 (18%) | 0.64 |
| Ischemic heart disease | 45 (9.1%) | 24 (7.4%) | 21 (12%) | 0.12 |
| Heart failure | 38 (7.7%) | 27 (8.4%) | 11 (6.4%) | 0.53 |
| Stroke | 27 (5.4%) | 15 (4.6%) | 12 (6.9%) | 0.39 |
| COPD | 58 (12%) | 33 (10%) | 25 (14%) | 0.21 |
| Asthma | 30 (6.0%) | 22 (6.8%) | 8 (4.6%) | 0.44 |
| Liver cirrhosis | 8 (1.6%) | <5 | <5 | 0.46 |
| **Medications at presentation** |  |  |  |  |
| ACE inhibitors or ARBs | 129 (26%) | 89 (28%) | 40 (23%) | 0.33 |
| Corticosteroids | 73 (15%) | 48 (15%) | 25 (14%) | >0.99 |
| Immunosuppressants | 15 (3.0%) | 9 (2.8%) | 6 (3.5%) | 0.88 |

Values are No. (%) or median [inter-quartile range].

Abbreviations: ACE = angiotensin converting enzyme; ARB = angiotensin receptor blockers; COPD = chronic obstructive pulmonary disease

† Scottish Index of Multiple Deprivation (SIMD) quintiles

‡ Comparison between patients with COVID-19 versus those with alternative diagnosis

**eTable 3.** Virology, laboratory tests at presentation stratified according to whether the diagnosis of COVID-19 was confirmed or probable

|  | **All**  **COVID-19**  (n = 496) | **Confirmed COVID-19**  (n = 323) | **Probable**  **COVID-19**  (n = 173) | **P-value*** |
| --- | --- | --- | --- | --- |
| Positive index PCR test | 255 (51%) | 255 (79%) | 0 (0%) | <0.001 |
| White cell count, x10^9^/L | 8.1 [6.0-11.8] | 7.8 [5.8-11.2] | 9.1 [6.4-12.3] | 0.016 |
| Lymphocyte count, x10^9^/L | 1.14 [0.78-1.61] | 1.08 [0.72-1.52] | 1.22 [0.90-1.79] | 0.003 |
| Neutrophil count, x10^9^/L | 5.8 [4.0-8.9] | 5.8 [3.9-8.5] | 5.9 [4.2-9.5] | 0.150 |
| C-reactive protein, mg/dL | 52 [13-123] | 58 [18-128] | 40 [8-105] | 0.005 |
| ALT, IU/L | 22 [14-36] | 22 [14-36] | 21 [15-34] | 0.530 |
| Impaired renal function | 158 (32%) | 110 (34%) | 48 (28%) | 0.180 |

Values are No. (%) or median [inter-quartile range].

Abbreviations: ALT = alanine transaminase; PCR = polymerase chain reaction

Impaired renal function = estimated glomerular filtration rate of <60 mL/min

* Comparison between patients with confirmed COVID-19 versus those with probable COVID-19

**eTable 4.** Use of serial testing in patients with suspected COVID-19

|  | **All**  **n=1368** | **Confirmed or probable COVID-19**  **n=496** | **Confirmed COVID-19** | | **Probable COVID-19**  **n=173** | **Alternative diagnosis**  **n=872** |
| --- | --- | --- | --- | --- | --- | --- |
|  |  |  | Index test positive  n=255 | Index test negative  n=68 |  |  |
| Serial testing | 815 (59.6) | 353 (71.2) | 168 (65.9) | 65 (95.6) | 120 (69.4) | 462 (53.0) |
| Number of tests per patient | 2 [1-3] | 2 [1-4] | 2 [1-5] | 4 [2-6] | 2 [1-3] | 2 [1-3] |
| Days between first and second serial test | 5.2 [1.1-15.0] | 4.6 [1.0-8.1] | 6.8 [4.0-8.6] | 1.7 [0.8-10.9] | 1.2 [0.9-4.6] | 6.1 [1.1-21.8] |

Values are No. (%) or median [IQR].

**eTable 5.** Diagnostic performance of the index and serial combined nasal and throat swab for the secondary outcome of a diagnosis of confirmed COVID-19 on serial testing

| **Serial tests** | **True negative** | **False negative** | **True positive** | **False positive** | **Sensitivity**  **(95% CI)** | **Negative predictive value**  **(95% CI)** | **Positive predictive value**  **(95% CI)** | **Specificity**  **(95% CI)** |
| --- | --- | --- | --- | --- | --- | --- | --- | --- |
| 1 (index test) | 1041 | 68 | 255 | 4 | 78.9 (76.7-81.0) | 93.9 (92.5-95.0) | 98.5 (97.7-99.0) | 99.6 (99.1-99.8) |
| 2 | 574 | 26 | 207 | 10 | 88.8 (86.5-90.8) | 95.7 (94.0-96.9) | 95.4 (93.7-96.6) | 98.3 (97.1-99.0) |
| 3 | 297 | 14 | 158 | 13 | 91.9 (89.1-94.0) | 95.5 (93.3-97.0) | 92.4 (89.7-94.4) | 95.8 (93.6-97.3) |
| 4 | 173 | 5 | 122 | 10 | 96.1 (93.3-97.7) | 97.2 (94.7-98.5) | 92.4 (88.9-94.9) | 94.5 (91.4-96.6) |
| 5 | 116 | 2 | 93 | 7 | 97.9 (95.0-99.1) | 98.3 (95.6-99.4) | 93.0 (88.8-95.7) | 94.3 (90.4-96.7) |
| 6 | 73 | 0 | 74 | 7 | 100.0 (97.6-100.0) | 100.0 (97.6-100.0) | 91.4 (85.9-94.8) | 91.2 (85.7-94.8) |
| 7 | 56 | 0 | 54 | 7 | 100.0 (96.8-100.0) | 100.0 (96.8-100.0) | 88.5 (81.5-93.1) | 88.9 (81.9-93.4) |
| 8 | 37 | 0 | 42 | 5 | 100.0 (95.6-100.0) | 100.0 (95.6-100.0) | 89.4 (81.0-94.3) | 88.1 (79.5-93.4) |

**eFigure 1.** Stack plot of the number of RT-PCR tests performed stratified according to whether the test was positive (red) or negative (blue).

**eFigure 2.** Heat map of RT-PCR testing in patients with confirmed COVID-19 stratified according to whether the index test was negative (a) or positive (b).

**
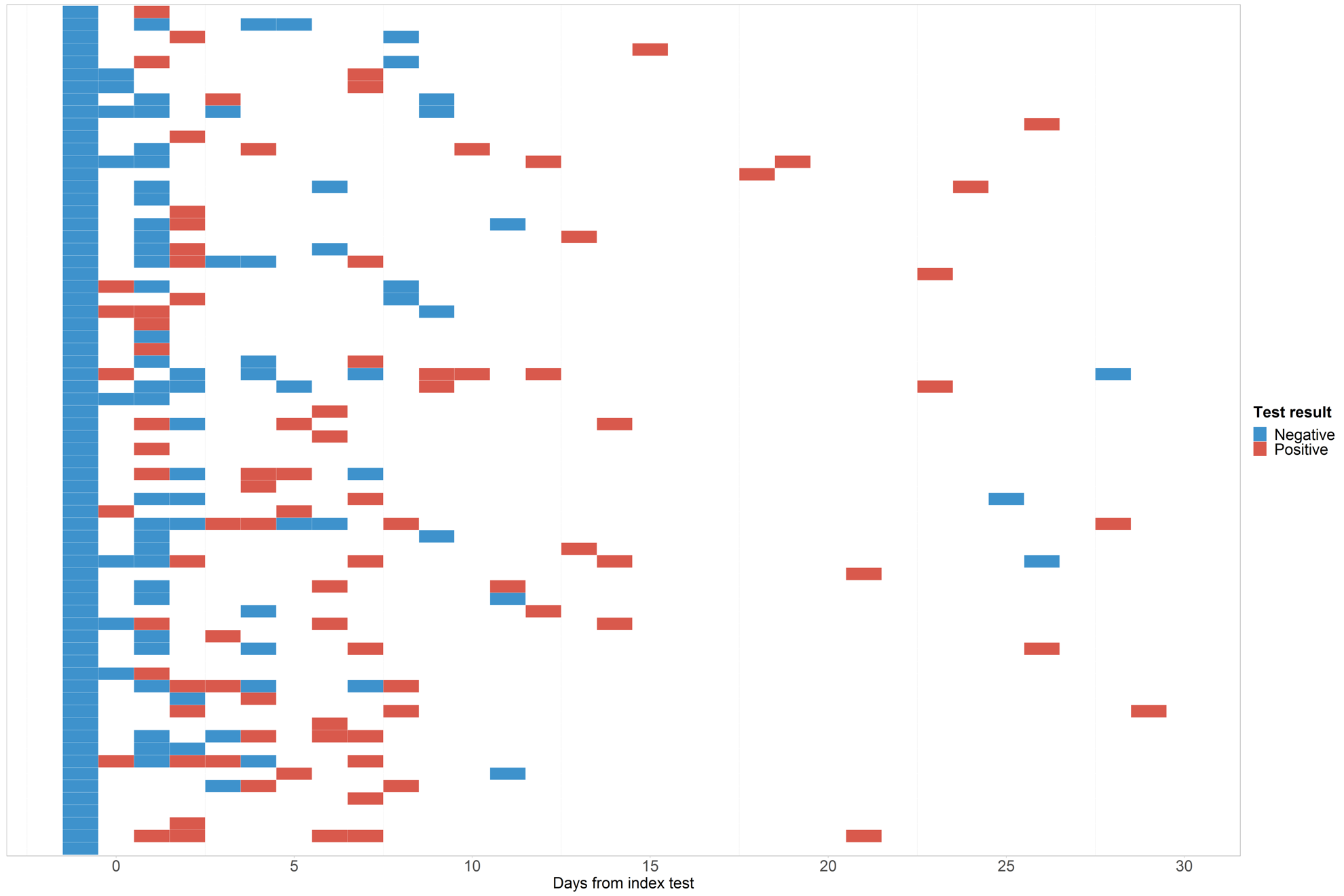
(a)**

**(b)**

**
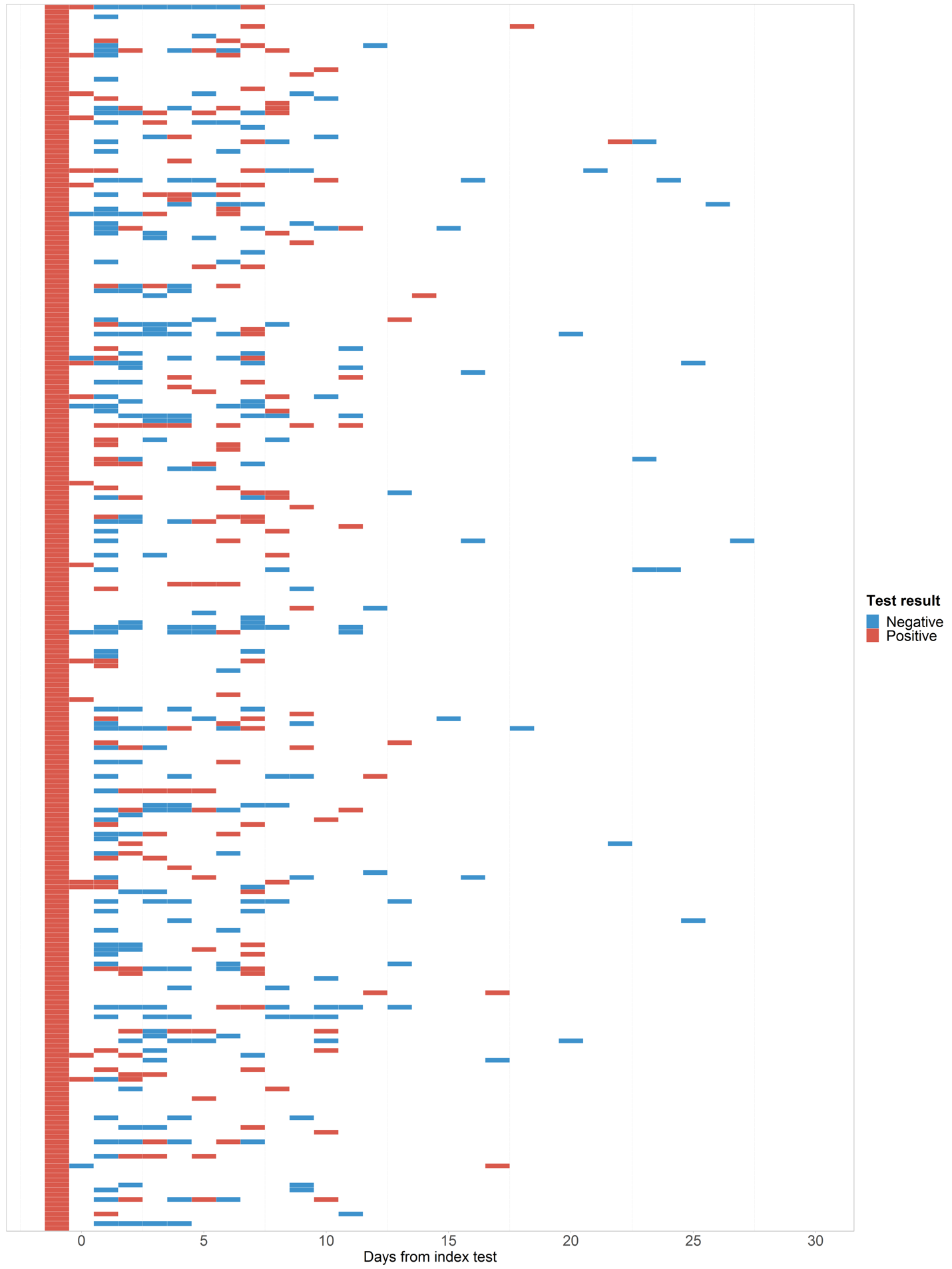
**

**eFigure 3.** Sensitivity of serial testing using the combined nasal and throat swab for the primary (confirmed and probable COVID-19) and secondary (confirmed COVID-19) outcome in patients who were tested at least four times

**eFigure 4.** Negative predictive value of serial testing using the combined nasal and throat swab for the primary (confirmed and probable COVID-19) and secondary (confirmed COVID-19) outcome in patients who were tested at least four times

**eFigure 5.** Forest plot of the (a) sensitivity and (b) negative predictive value of the index combined nasal and throat swab for a diagnosis of confirmed COVID-19 stratified by subgroups

## (a)


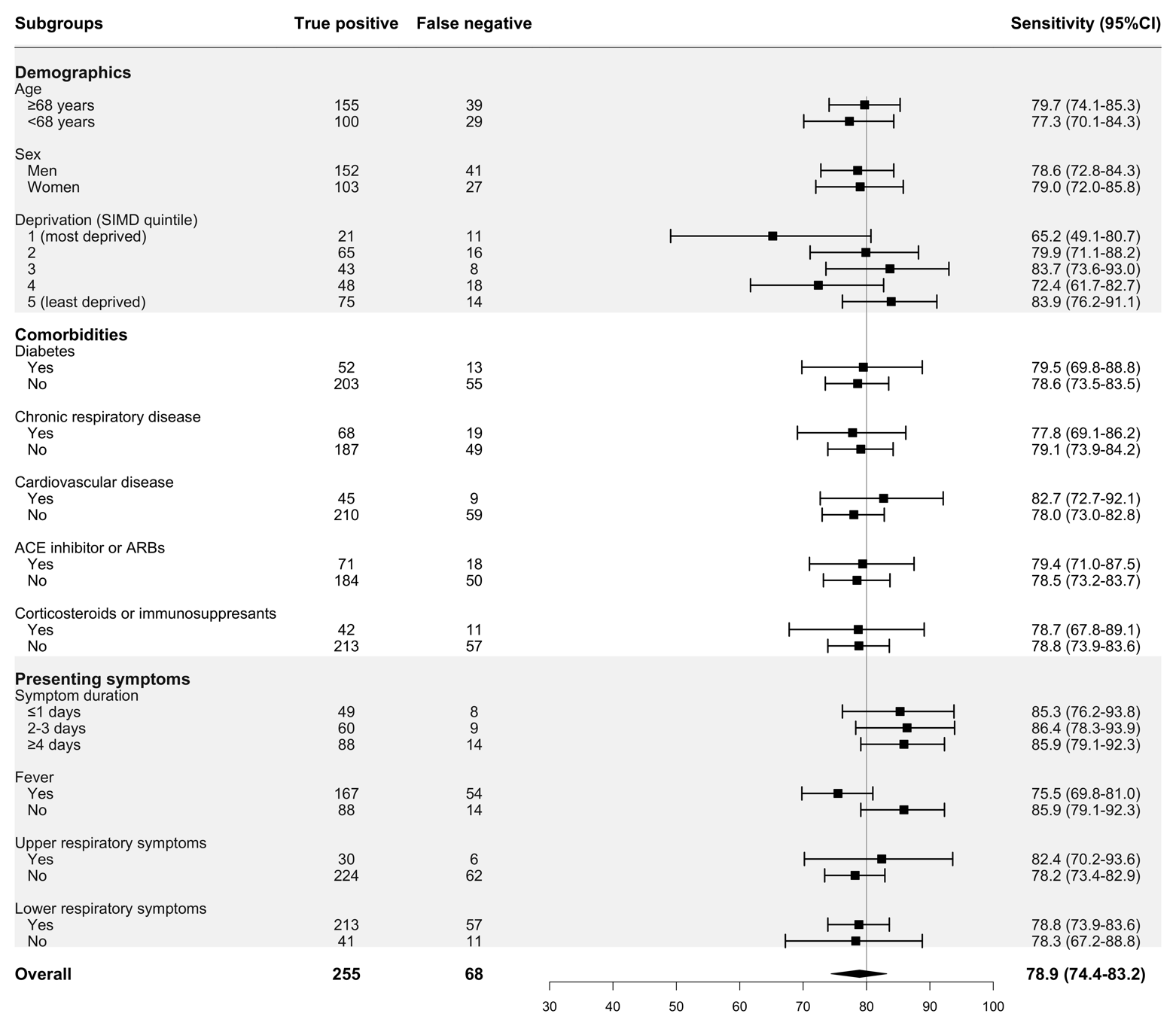


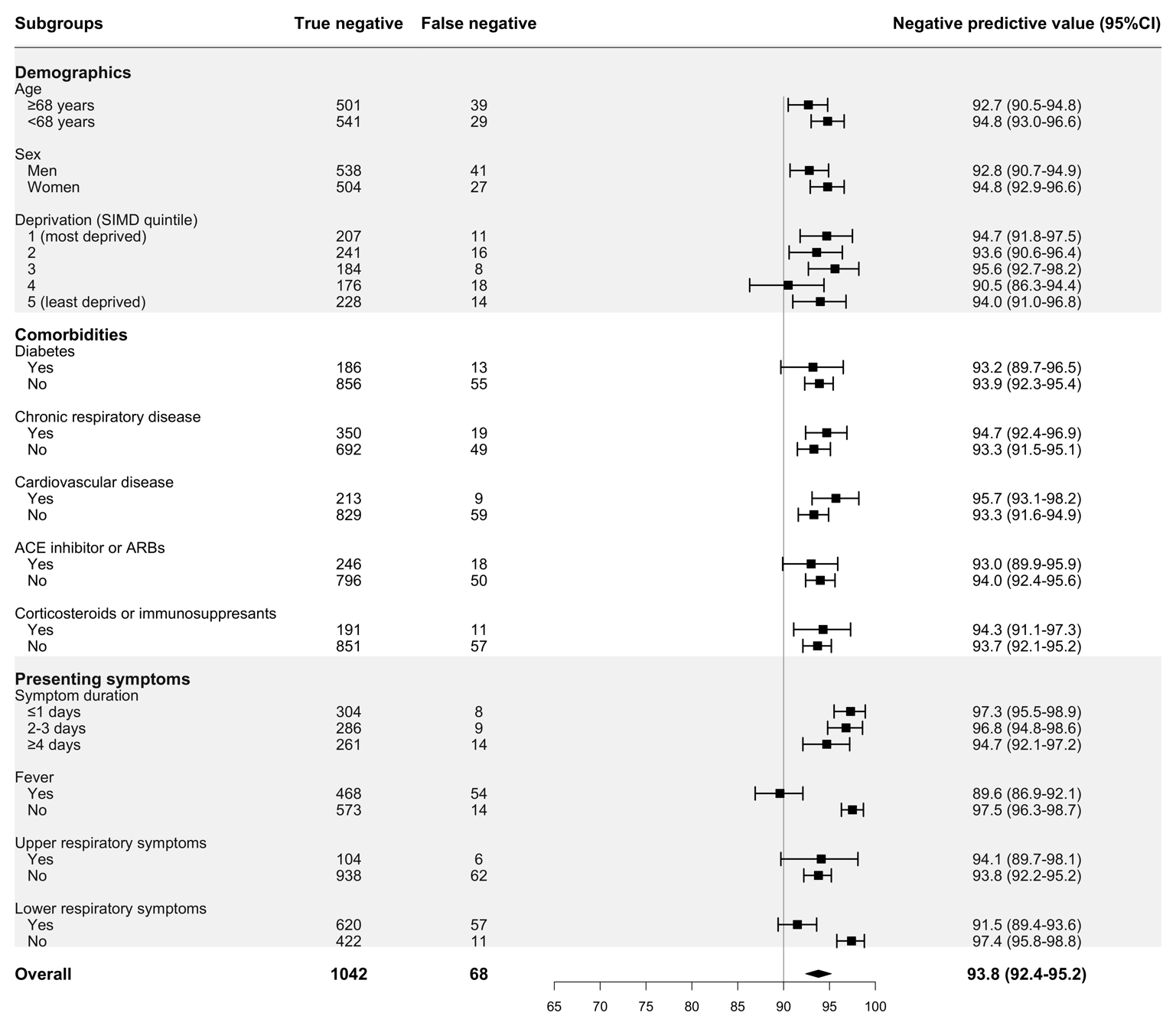
**(b)**
